# Putative extremely high rate of proteome innovation in lancelets might be explained by high rate of gene prediction errors

**DOI:** 10.1038/srep30700

**Published:** 2016-08-01

**Authors:** László Bányai, László Patthy

**Affiliations:** 1 Institute of Enzymology, Research Centre for Natural Sciences, Hungarian Academy of Sciences, Budapest, P.O. Box 286, H-1519, Hungary

## Abstract

A recent analysis of the genomes of Chinese and Florida lancelets has concluded that the rate of creation of novel protein domain combinations is orders of magnitude greater in lancelets than in other metazoa and it was suggested that continuous activity of transposable elements in lancelets is responsible for this increased rate of protein innovation. Since morphologically Chinese and Florida lancelets are highly conserved, this finding would contradict the observation that high rates of protein innovation are usually associated with major evolutionary innovations. Here we show that the conclusion that the rate of proteome innovation is exceptionally high in lancelets may be unjustified: the differences observed in domain architectures of orthologous proteins of different amphioxus species probably reflect high rates of gene prediction errors rather than true innovation.

Lancelet is the extant representative of basal chordates (Cephalochordate), which diverged from other chordate lineages (urochordates and vertebrates) about 550 Myr ago^1^. Lancelets are widely regarded as living fossils as they retained a body plan and morphology most similar to the fossil chordates from the early Cambrian (*Yunnanozoon lividum* and *Haikouella lanceolatum*) and from the middle Cambrian (*Pikaia gracilens*)^2^,^3^,^4^,^5^.

Extreme conservation of the morphology of lancelets is also supported by the fact that although *Branchiostoma floridae*, *Branchiostoma lanceolatum* and *Branchiostoma belcheri* diverged ~100–200 Myr ago they are remarkably similar, suggesting that their networks of developmental genes were practically unchanged. In harmony with this assumption the expression pattern of genes implicated in different developmental functions of *B. lanceolatum* and *B. floridae* is conserved: the different species of amphioxus appear to be very similar, not only morphologically, but also in the genetic programs directing the development of their structural features^6^.

A recent study, however, has suggested that the proteomes of the Chinese amphioxus, *B. belcheri* and the Florida amphioxus, *B. floridae* are markedly different^7^. In this study Huang *et al.* have compared presence–absence status of protein domain combinations in proteins of various species and concluded that − in contrast to vertebrates − lancelets evolved rapidly and continuously, ultimately acquiring threefold more domain combinations than any vertebrate. According to their estimates lancelets gained new domain pairs at a rate of >10 per Myr, which is 10- to 100-fold higher than that observed in other metazoan lineages^7^. The authors hypothesized that high diversity and continued activity of transposable elements in the lancelet lineage increases the rate of exon shuffling and the rate of creation of novel protein domain combinations, explaining the high rate of protein innovation.

Interestingly, the unusually high rate of domain architecture alteration is not accompanied by a high amino acid substitution rate. Out of the orthologs present both in *B. belcheri* and *B. floridae* as well as thirteen other metazoan species the authors have selected a set of 729 protein-coding genes and compared the rate of their divergence in the different lineages. In harmony with earlier data of Delsuc *et al.*^1^ and Putnam *et al.*^8^ the authors have found that lancelets show fewer amino-acid substitutions (shorter branches) than urochordates and vertebrates^7^. Thus, in this respect, lancelets are similar to other living fossils in as much as they are usually characterized by very slow rates of molecular evolution. It is generally accepted that the slow rate of nucleotide substitution offers an explanation as to why the morphology of coelacanths has evolved so slowly over the past 400 million years and why the morphology of turtles was conserved from the Triassic to the present^9^,^10^,^11^. Low substitution rate in a living fossil also seems to hold for the lophotrochozoan *Lingula*. In fact, the abundance of *Lingula* fossils from the Silurian, with morphology very similar to that of extant species, inspired Darwin with the idea of ‘living fossils’. Luo *et al.*^12^ have shown recently that *Lingula* genes associated with basic metabolism, such as ribonucleoprotein complex biogenesis and RNA processing, show the slowest evolutionary rate among lophotrochozoans^12^.

Whereas the low rate of amino acid substitution appears to be in harmony with the low rate of morphological evolution in lancelets, the exceptionally high rate of creation of novel proteins (with novel domain combinations) would suggest that major changes in the complexity of the lancelet proteomes are not coupled to significant changes in phenotype. It must be emphasized that this seems to contradict the observation that high rates of creation of novel proteins are usually associated with major evolutionary innovations such as those that accompanied the appearance of metazoa and vertebrates^13^.

In the present work we provide a possible explanation for this contradiction. We suggest that the conclusion that the rate of protein innovation is exceptionally high in lancelets may be unjustified: we demonstrate that the differences in domain architecture of orthologous proteins of the two amphioxus species reflect gene prediction errors rather than true innovation.

## Results and Discussion

### Gene models of *B. belcheri* and *B. floridae* suffer from high rates of gene prediction errors

Gene models of *B. belcheri* were predicted by Huang *et al.*^7^ by integrating the results obtained by *de novo* gene prediction, homology-based and transcriptome-based prediction using cDNA alignments, protein alignments, RNA-seq alignments, *ab initio* data sets and RNA-seq-based predictions^14^,^15^,^16^,^17^,^18^. On the basis of ~300 million EST read pairs, the authors predicted 30,392 protein-coding genes in the Chinese lancelet genome, of which only 18,167 were found to have orthologs in the Florida lancelet. It should be noted that the genome of the Florida lancelet^8^ was estimated to contain only ~21,900 protein-coding loci, based on gene models created by standard methods, using about 480,000 ESTs, integrating homology and *ab initio* gene prediction methods.

Taken at face value, this difference in the number of predicted genes of the two lancelets is surprising since it would suggest that the Chinese lancelet invented or that the Florida lancelet has lost thousands of unique protein-coding genes. Since this dramatic difference in the number of unique genes is not reflected in major phenotype differences of the two species, a more plausible explanation is that the number of genes of *B. floridae* is underpredicted and/or that the number of genes of *B. belcheri* is overpredicted.

It is important to point out that the draft genomes of *B. belcheri* and *B. floridae* are only available at the scaffold level, therefore genes may be fragmented onto multiple individual contigs and this is known to lead to significant errors in the apparent number of genes^19^. In addition to this problem of draft genomes, prediction of protein-coding genes is not trivial even if we have finished genome assemblies. The ENCODE Genome Annotation Assessment Project^20^ has clearly shown that − in the case of intron-rich genomes of higher eukaryotes − prediction of the correct structure of protein-coding genes remains a difficult task. In the EGASP study a set of well annotated ENCODE sequences were blind analyzed with different gene finding programs and the predictions obtained were analyzed to evaluate how well they reproduce the annotations^20^. None of the strategies produced perfect predictions but prediction methods that rely on experimental evidence (protein, mRNA and EST sequences) were generally more accurate. Nevertheless, such analyses have shown that the exact genomic structure of protein-coding genes of vertebrates is correctly predicted for only ~60% of the genes^21^.

The problem of gene prediction errors appears to be even more severe in the case of lancelet genomes: our analysis of the predicted proteomes of various metazoa with MisPred tools have shown that the rate of misprediction of lancelet genes is significantly higher than in the case of vertebrate genomes^22^,^23^. Accordingly, in the case of the genomes of *B. floridae* and *B. belcheri* a high proportion of gene models is mispredicted.

### Comparison of the predicted proteomes of *B. belcheri* and *B. floridae* is likely to overestimate the rate of protein innovation

Huang *et al.*^7^ have included the predicted proteins of *B. belcheri and B. floridae* in an analysis of the presence–absence status of various domain combinations in different metazoa species. In harmony with earlier data^13^,^24^, they have concluded that the rates of gaining new domain combinations were elevated during early vertebrate evolution but reduced in jawed vertebrates. Surprisingly, their analyses suggested that lancelets evolved rapidly and continuously; according to the authors’ estimate lancelets gained new domain-combinations at 10- to 100-fold higher rate than that normally observed in metazoans^7^.

In our earlier work we have pointed out that in genome-scale studies of protein domain architecture evolution it should not be ignored that a significant proportion of metazoan sequences is mispredicted and that this may seriously affect the validity of the conclusions drawn from such analyses^25^. We have emphasized that when predicted proteomes are compared the contribution of gene prediction errors to domain architecture differences of orthologs may be far greater than those due to true gene rearrangements^25^.

### Domain architecture differences of orthologs of *B. belcheri* and *B. floridae* reflect errors of gene prediction

To estimate the actual contribution of gene prediction errors to the exceptionally high (apparent) rate of protein innovation in lancelets, we have randomly selected 100 proteins of the *B. belcheri* proteome (the Branchiostoma.belcheri_HapV2_proteins.fa dataset) that contained at least two Pfam-A domains and identified their orthologs (if any) in the *B. floridae* proteome by the reciprocal best-hit method. The rationale of this approach is that when sequences from two complete proteomes are compared orthologs give reciprocal best hits, i.e. protein sequence A of species 1 finds protein sequence A’ of species 2 as its best hit and *vice versa*. The most important limitation of the reciprocal best match approach is that it may lead to erroneous conclusions if the proteomes compared are incomplete: paralogs or epaktologs may be identified as each other’s best match, instead of the missing orthologs^26^,^27^. Another limitation of the reciprocal best match approach is that if, following divergence of species, the orthologous genes were duplicated in one or both species, the approach will identify just one member of the co-orthology groups as the reciprocal best match. It must be pointed out that in this case the conclusion of orthology will not be erroneous but fails to identify all co-orthologs.

In the present case these dangers appear to be significant; since the *B. floridae* genome was predicted to contain far fewer genes (~21,900 protein-coding genes) than *B. belcheri* (30,392 protein-coding genes) and since one-third of *B. belcheri* proteins had no ortholog in the *B. floridae* proteome^7^, it seems likely that the *B. floridae* proteome is less complete than the *B. belcheri* proteome. Since the mean identity of orthologous proteins of the Chinese and Florida lancelets was calculated to be 81.2%, in the present work we used a cut off-value of 60% amino acid sequence identity as the minimum requirement for orthologs. Using these criteria we have found that 93 of the selected *B. belcheri* proteins had orthologs in the predicted proteome of *B. floridae* (Table 1 and Supplementary Tables S1–S3). The domain architectures of the orthologs thus selected were compared as described previously^25^.

As shown in Table 1, in the case of release 1, release 2 and release 3 of *Branchiostoma belcheri* sequences 40, 49 and 42 (43%, 53% and 45%) of the 93 ortholog-pairs of the two lancelet species had non-identical domain architectures. The rate of domain architecture differences in Chinese and Florida lancelets thus appears to be an order of magnitude higher than that observed in the case of high quality UniProtKB/Swiss-Prot sequences of Metazoan species (*Homo-Mus*: 1.1%; *Homo-Gallus*: 3.00%; *Homo-Xenopus*: 0.9%; *Homo-Danio*: 2.1%; *Homo-Drosophila*: 4.8%; *Homo-Caenorhabditis*: 5.9%)^25^.

In cases where orthologous proteins of *B. belcheri* and *B. floridae* differed in domain architecture our working hypothesis was that domain architecture deviation is more likely to reflect errors in gene prediction than true innovation^25^. In order to distinguish the ‘correct’ and ‘erroneous’ members of the ortholog pair, we have compared their domain architectures with those of their orthologs present in the high quality Swiss-Prot database: the protein with the same domain architecture as those of orthologous Swiss-Prot entries was judged to be correctly predicted. Out of the 100 proteins selected 74 had orthologs in the Swiss-Prot database (Table 1 and Supplementary Tables S1–S3). In 29 cases the domain architectures of proteins from both *B. belcheri* and *B. floridae* were identical with those of their Swiss-Prot orthologs, but in 15 cases only the *B. belcheri* protein, in 8 cases only the *B. floridae* had the same domain architecture as their Swiss-Prot orthologs. Note that in 22 cases both orthologs proved to deviate from the domain architecture of their orthologs in the Swiss-Prot database.

To decide whether the domain architecture deviation reflects an error in gene prediction (and not true change in gene structure) we have subjected the ‘erroneous’ member(s) of the orthologous pair to the FixPred protocol to correct the prediction errors. FixPred protocol attempts to correct erroneous sequences in several steps, starting with the simplest solutions (finding experimental evidence for the correct sequence version in protein or cDNA and EST databases), progressing to more time-consuming gene-predictions^28^.

Our analyses have shown that the differences in the domain architecture of orthologs of *B. belcheri* and *B. floridae* reflect errors of gene prediction, as evidenced by the fact that in all cases the FixPred approach found genomic evidence for the identity of the domain architectures of orthologs of the two lancelet species (Table 1).

The role of gene prediction errors is underlined by the fact that reannotation of improved genome assemblies failed to decrease the rate of misprediction (see Table 1 and Supplementary Table S3). In fact, our analyses of the three different datasets (see Supplementary Table S3) have identified several cases where the more recent releases replaced correct predictions by erroneous predictions. For example, in release 1 protein 246010_PRF0, an ortholog of vertebrate matrix metalloproteinase-16, was correctly predicted whereas in release 3 it was “fused” to an ortholog of proton-coupled folate transporter (see Supplementary Table S3).

Nevertheless, our data suggest that in the long run improved annotation of lancelet genomes would reduce domain architecture differences of ortholog-pairs of lancelet species to a level typical of other Metazoa. Since Chinese and Florida lancelets diverged about 120 million years ago, roughly at the same time as the divergence of major mammalian lineages, it seem likely that the rate of domain architecture differences of lancelet species will be reduced to levels similar to those observed in comparison of *Homo-Mus* orthologs^25^.

Here we illustrate our analysis with a few prototypical examples selected from the lists presented in Supplementary Tables S1, S2 and S3.

#### WFIKKN protein

**Figure 1:**
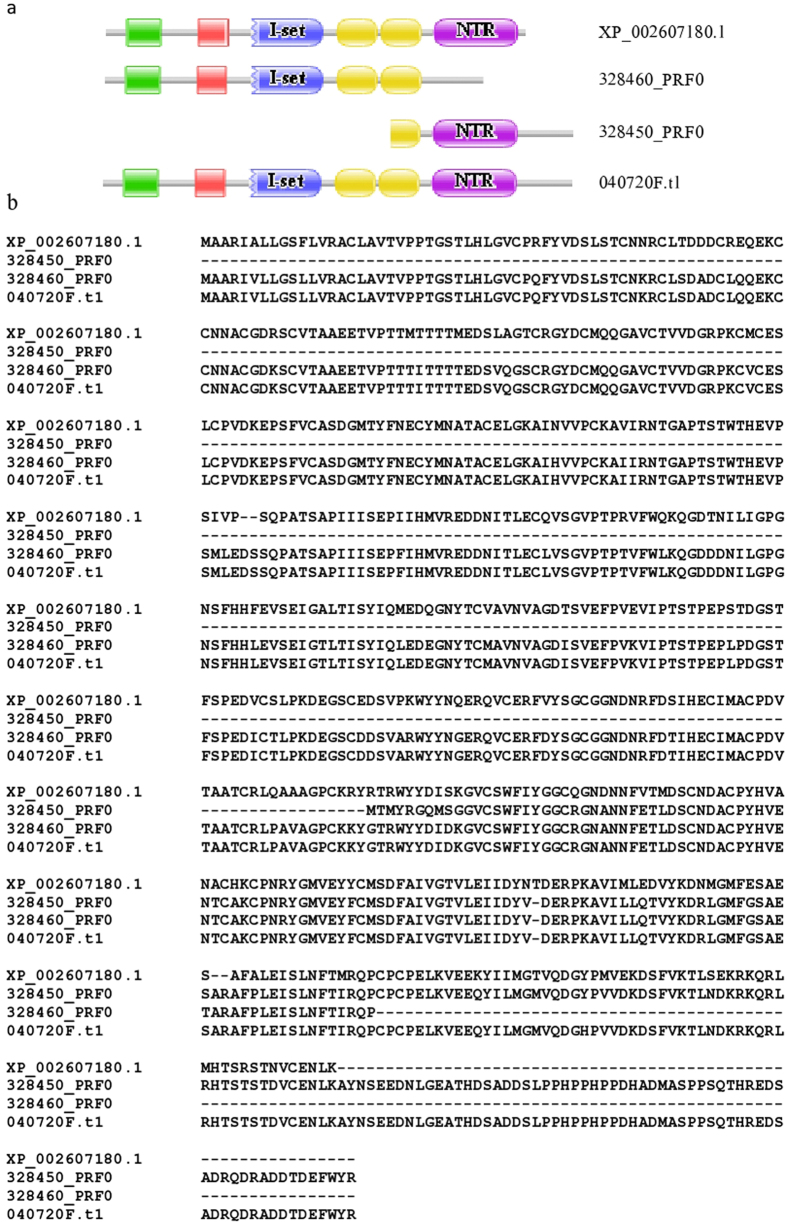
The WFIKKN gene was mispredicted in the genome annotation of *B. belcheri*. Predicted proteins 328450_PRF0 and 328460_PRF0 proteins of the dataset Branchiostoma.belcheri_HapV2_proteins.fa were shown to be orthologous with different parts of XP_002607180.1, the WFIKKN protein of *B. floridae*, but Branchiostoma.belcheri_v18h27.r3_ref_protein.fa dataset contains a single, full-length ortholog of XP_002607180.1, protein 040720F.t1. (**a**) Domain architectures of 328450_PRF0, 328460_PRF0, XP_002607180.1 and 040720F.t1. (**b**) Alignment of the sequences of 328450_PRF0, 328460_PRF0, XP_002607180.1 and 040720F.t1. Note that the apparent difference between the domain architectures of WFIKKN orthologs of *B. belcheri* (proteins 328450_PRF0 and 328460_PRF0) and *B. floridae* (XP_002607180.1) reflected an error in gene prediction and not true change in gene structure. Color code for Pfam-A domains: WAP – green; Kazal – red; Immunoglobulin – blue; Kunitz – yellow; NTR – purple.

The 328450_PRF0 protein of *B. belcheri* was found to be orthologous with the C-terminal part of XP_002607180.1 of *B. floridae*, whereas the reciprocal best match search revealed that protein 328460_PRF0 of *B. belcheri* (encoded by a neighboring region on the same scaffold of the *B. belcheri* genome) is orthologous with the N-terminal part of XP_002607180.1 (Fig. 1).

Comparison of the domain architectures of 328450_PRF0, 328460_PRF0 and XP_002607180.1 with those of their orthologs in the Swiss-Prot database revealed that XP_002607180.1 of *B. floridae* has a domain architecture typical of WFIKKN proteins of vertebrates. These large extracellular multidomain proteins appeared in chordates; they consist of WAP-, Follistatin/Kazal-, Immunoglobulin, two Kunitz-type domains and an NTR-domain^29^,^30^,^31^.

The difference between the domain architectures of WFIKKN orthologs of *B. belcheri* and *B. floridae* thus reflects an error of the prediction of the *B. belcheri* proteome. It should be noted that the Branchiostoma.belcheri_v18h27.r3_ref_protein.fa dataset does not suffer from this error; it contains a single, full-length ortholog of XP_002607180.1 (protein 040720F.t1 in this dataset).

#### Calcium-binding mitochondrial carrier protein

**Figure 2:**
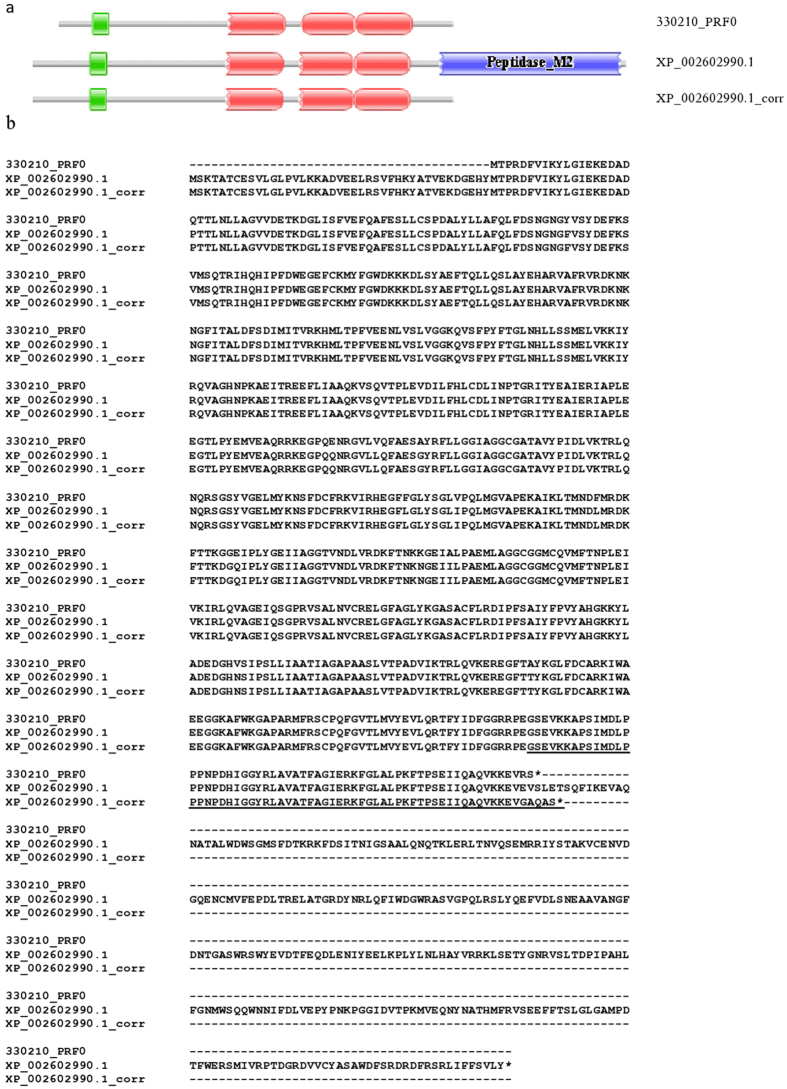
The gene of calcium-binding mitochondrial carrier protein is mispredicted in the genome annotation of *B. floridae*. (**a**) The calcium-binding mitochondrial carrier protein 330210_PRF0 of *B. belcheri* is an ortholog of the *B. floridae* protein XP_002602990.1, but the latter contains a C-terminal extension with a Peptidase_M2 domain homologous with angiotensin-converting enzymes. Tblastn searches of the database of *B. floridae* ESTs with the XP_002602990.1 protein identified ESTs (BI386388.1 and BW904813) that indicate that the presence of the Peptidase_M2 domain in XP_002602990.1 reflects an error in gene prediction, permitting the correction of this sequence (XP_002602990.1_corr). (**b**) Alignment of the sequences of 330210_PRF0, XP_002602990.1 and XP_002602990.1_corr. Residues supported by both ESTs (BI386388.1 and BW904813 are underlined. Note that the apparent difference between the domain architectures of calcium-binding mitochondrial carrier protein orthologs of *B. belcheri* and *B. floridae* reflected an error in gene prediction and not true change in gene structure. Color code for Pfam-A domains: EF-hand – green; Mito_carr – red; Peptidase_M2 – blue.

The 330210_PRF0 protein of *B. belcheri* is a full-length orthologue of calcium-binding mitochondrial carrier proteins, containing an EF-hand domain and three Mito_carr domains (Fig. 2). The 330210_PRF0 protein and its *B. floridae* orthologue (XP_002602990.1) are 98% identical in the aligned region, but the *B. floridae* protein has a long C-terminal extension with a Peptidase_M2 domain homologous with angiotensin-converting enzyme.

There are several reasons why the presence of a Peptidase_M2 domain in XP_002602990.1 was likely to reflect gene prediction error and not innovation. First, Peptidase_M2 domains are usually present in extracellular proteins, whereas Mito_carr domains are restricted to the intracellular space; their co-occurrence violates one of the basic dogmas that MisPred uses to detect gene prediction errors^22^,^23^. Second, the average length of Peptidase_M2 domains is 470 amino acid residues with little variation^32^, but it is only ~230 residue in the *B. floridae* protein. Such drastic truncation of a globular domain violates another of the basic dogmas that MisPred employs to detect gene prediction errors^22^,^23^. The key evidence that XP_002602990.1 is mispredicted comes from analyses of ESTs. Tblastn searches of the database of *B. floridae* ESTs with the XP_002602990.1 protein identified several ESTs (FE578243.1, BI386388.1, BW799678.1 and BW904813.1) that originate from the putative ‘fusion’ region but they argue against this fusion: they match either the N-terminal or the C-terminal fusion partners part but not both parts of the chimeric XP_002602990.1 protein. ESTs BI386388.1 and BW904813 identified the stop codon of the calcium-binding mitochondrial carrier protein of *B. floridae,* permitting the correction of its sequence (Fig. 2). The difference between the domain architectures of calcium-binding mitochondrial carrier proteins of *B. belcheri* and *B. floridae* thus reflects an error of the prediction of the *B. floridae* proteome and not true innovation.

#### Agrin

Protein 127590_PFF0 of *B. belcheri* is orthologous with the N-terminal part of the *B. floridae* protein, XP_002614044.1, whereas the reciprocal best match search revealed that protein 127600_PFF0 of *B. belcheri* (encoded by a neighboring region on the same scaffold of the B. belcheri genome) is orthologous with the C-terminal part of XP_002614044.1.

Comparison of the domain architectures of 27590_PFF0, 127600_PFF0 and XP_002614044.1 with those of their orthologs in the Swiss-Prot database revealed that the domain architecture of XP_002614044.1 is typical of agrin, a basement membrane protein highly conserved both in protostomes and deuterostomes^33^, suggesting that its ‘fission’ in *B. belcheri* reflects an error in gene prediction rather than true change in domain organization. It should be noted that the Branchiostoma.belcheri_v18h27.r3_ref_protein.fa dataset is exempt from this error: predicted protein 212090F.t4 is a full-length agrin.

#### Vacuolar protein sorting-associated protein 13D

**Figure 3:**
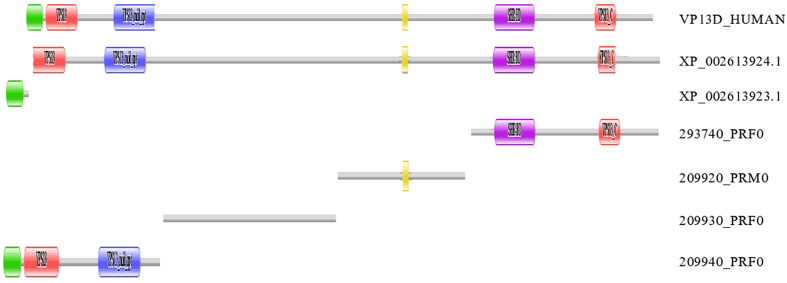
The genes of vacuolar protein sorting-associated protein 13D are mispredicted in the genome annotations of both *B. belcheri* and *B. floridae*. Vacuolar protein sorting-associated protein 13D is a large, highly conserved multidomain protein containing Chorein_N, VPS13, VPS13_mid_rpt, UBA, SHR-BD and VPS13_C domains (represented here by VP13D_HUMAN). Genome annotation, however, identified four different parts of the VP13D protein of *B. belcheri* and two different parts of the *B. florida* protein as independent proteins. Note that the apparent difference between the domain architectures of orthologous parts of vacuolar protein sorting-associated protein 13D from *B. belcheri* and *B. floridae* reflect an error in gene prediction and not true change in gene structure. Color code for Pfam-A domains: Chorein_N – green; VPS13 – red; VPS13_mid_rpt – blue; UBA – yellow; SHR-BD – purple; VPS13_C – pink.

The 293740_PRF0 protein of *B. belcheri* was found to be orthologous with the C-terminal part of XP_002613924.1 of *B. floridae* (Fig. 3). Iteration of reciprocal best match searches revealed that proteins 209920_PRM0, 209930_PRF0 are orthologous with the middle part of XP_002613924.1, whereas protein 209940_PRF0 is orthologous with the N-terminal part of XP_002613924.1. The 209940_PRF0 protein of *B. belcheri*, however, contains a region that is absent in XP_002613924.1; this region was found to be orthologous with protein XP_002613923.1 of *B. floridae*.

Searching the UniProt KB protein database with proteins 209920_PRM0, 209930_PRF0, 293740_PRF0, 209940_PRF0, XP_002613924.1 and XP_002613924.1 identified Vacuolar protein sorting-associated protein 13D as their best match. Vacuolar protein sorting-associated protein 13D is a highly conserved multidomain protein that is involved in targeting proteins to the vacuole; they contain Chorein_N, VPS13, VPS13_mid_rpt, UBA, SHR-BD and VPS13_C domains (Fig. 3).

In view of the high conservation of this domain architecture of Vacuolar protein sorting-associated protein 13D the most plausible explanation for its fragmentation in both *B. belcheri* and *B. floridae* is that it reflects errors of gene prediction. In the case of *B. belcheri,* one of the reasons why the VPS13D protein was fragmented is that gene fragments encoding the N-terminal (proteins 209920_PRM0, 209930_PRF0 and 209940_PRF0) and C-terminal parts (protein 293740_PRF0) of VPS13D were not located on a single scaffold. In the case of *B. floridae* all parts of the VPS13D protein are encoded on the same scaffold (BRAFLscaffold_1, NW_003101570.1), but genome annotation identified the N-terminal part as an independent protein. (Note that the Branchiostoma.belcheri_v18h27.r3_ref_protein.fa dataset also contains the four fragments of the VPS13D protein as ‘independent” proteins: proteins 257090F.t1, 257100F.t1, 257120F.t1 and 257130F.t1).

In other words, the fact that orthologous pair of proteins of *B. floridae* and *B. belcheri* derived from their VPS13D proteins differ markedly in domain architecture reflects errors in gene prediction in both species and not proteome innovation.

### Absence of orthologs of *B. belcheri* proteins in *B. floridae* may reflect errors of gene prediction

If a *B. belcheri* protein (containing two Pfam-A domains) had no orthologue in the *B. floridae* proteome but orthologs from other metazoa were present in the high quality Swiss-Prot database our working hypothesis was that this reflects an error of genome annotation rather than true loss of a gene. In such cases we subjected genomic sequences of *B. floridae* to gene prediction with GeneWise^34^ using the given *B. belcheri* protein as input to find evidence for the presence of the ‘missing’ gene.

**Figure 4:**
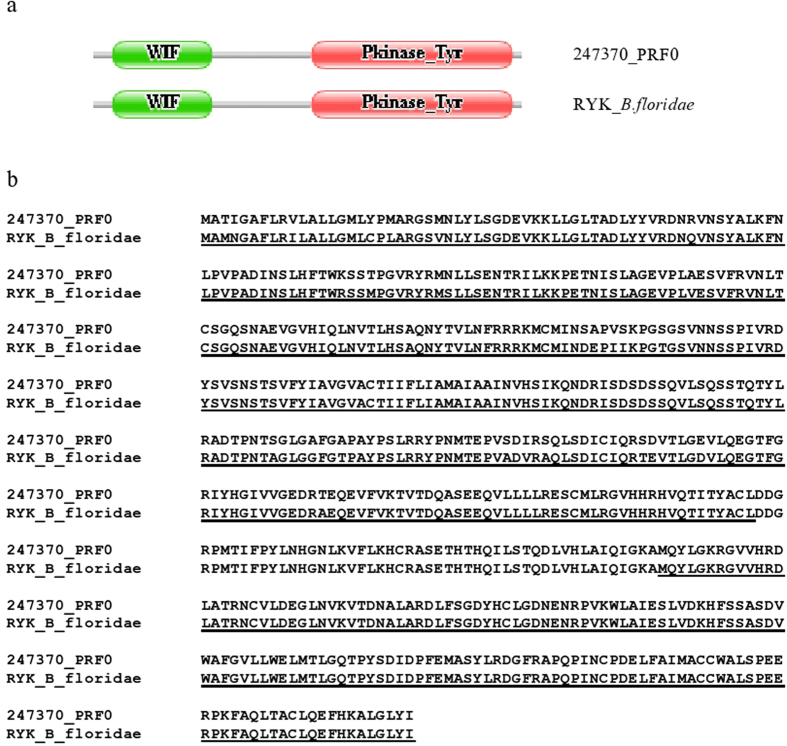
Evidence that a Ryk receptor tyrosine kinase gene is present *Branchiostoma floridae.* Ryk receptor tyrosine kinases, consisting of WIF domain (WIF) and a tyrosine kinase domain (Pkinase_Tyr) are present in all groups of Bilateria as well as in Cnidaria. Although the *B. belcheri* genome was predicted to encode a full-length Ryk receptor tyrosine kinase (247370_PRF0), no orthologue with the same domain architecture was found in the predicted proteome of *B. floridae*^14^. Our analysis of the *B. floridae* genome provided evidence for a Ryk gene (RYK_Bfloridae) that encodes a protein with the same domain organization as that of *B. belcheri* (**a**). Note that the predicted Ryk receptor tyrosine kinases of *B. belcheri* and *B. floridae* are 95% identical at the amino acid sequence level. In the alignment of the two sequences the residues supported by ESTs (BW781727.1, BW727057.1 and BW727057) are underlined (**b**).

This approach may be illustrated with the case of protein 247370_PRF0 of *B. belcheri* that has a unique domain architecture characteristic of Ryk receptor tyrosine kinases. Ryk receptor tyrosine kinases have a WIF domain in their extracellular ligand-binding region that serves to bind Wnts^35^,^36^. Ryk receptor tyrosine kinases are present in all groups of Bilateria as well as in Cnidaria^36^ therefore it was surprising that no ortholog was found in the predicted proteome of *B. floridae*. In harmony with the conservation of Ryk in Bilateria, analysis of the *B. floridae* genome and transcriptome provided evidence for a functional Ryk gene that encodes a protein with the same domain organization as that of *B. belcheri* (Fig. 4). Our analysis of the *B. floridae* genome with GeneWise using the 247370_PRF0 protein as input identified a region (BRAFLscaffold_191, NW_003101414.1) that contains the exons of a hypothetical Ryk receptor tyrosine kinase, permitting the construction of a gene model of the Ryk gene of *B. floridae*. Tblastn searches of the dataset of *B. floridae* ESTs with the predicted protein identified three ESTs (BW781727.1, BW727057.1 and BW727057) that provide full support for the gene model, confirming that *B. floridae* has a functional Ryk gene (Fig. 4). In other words, the fact that the WIF/Pkinase_Tyr domain combination was present in the predicted proteome of *B. belcheri* but not in that of *B. floridae* reflects an error in gene prediction (i.e. the gene model of Ryk is not represented in the predicted proteome of *B. floridae*).

### Some novel domain architectures apparently unique to *Branchiostoma* may reflect errors of gene prediction

Many of the *B. belcher*-*B. floridae* ortholog pairs listed in Supplementary Tables S1 and S2 have identical but unique domain architectures in as much as orthologous proteins with the same domain architecture are absent in the high quality Swiss-Prot database, either because the domain architecture of their Swiss-Prot orthologs are different or they have no orthologs in the Swiss-Prot database.

For example, the first category contains proteins that appear to be evolutionary precursors/variants of vertebrate-specific proteins (e.g. the neurotrypsin-related ortholog pair, 133320_PFF0 and XP_002610402.1 in Supplementary Table S1).

**Figure 5:**
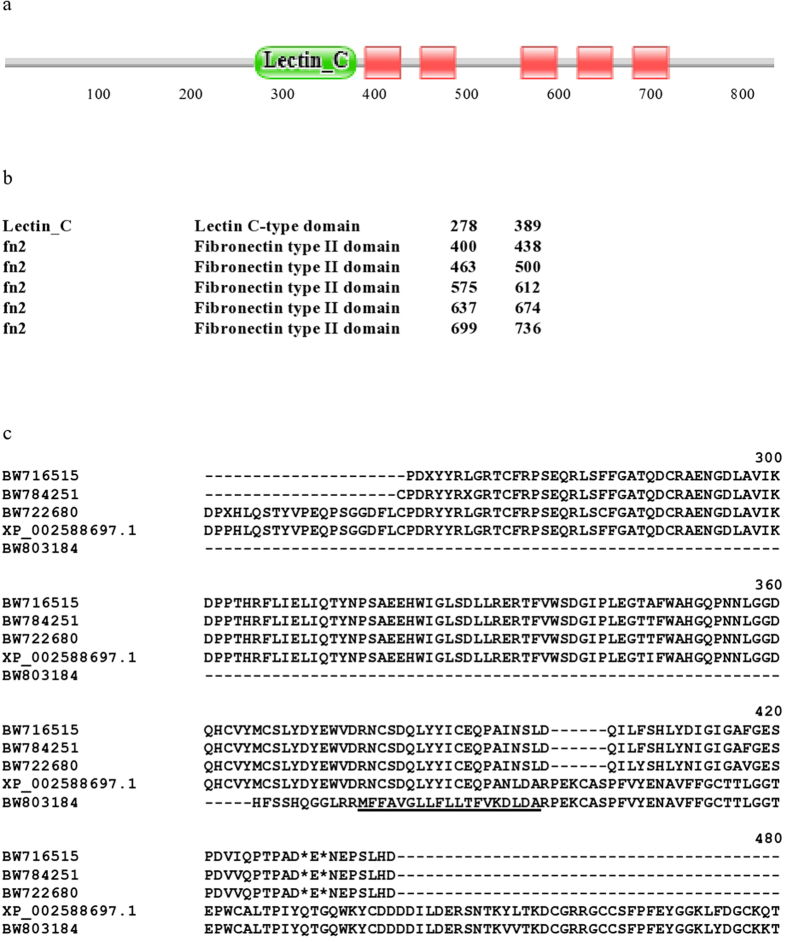
Evidence that a protein with a novel domain architecture present in the predicted proteomes of *B. belcheri* and *B. floridae* reflects errors of gene prediction and not true innnovation. Protein 018300_PFF0 of *B. belcheri* and its *B. floridae* ortholog, XP_002588697.1 contain a Lectin_C domain and five tandem fn2 domains. (**a**) pictogram of the domain architecture of XP_002588697.1. (**b**) positions of the various Pfam_A domains in the linear sequence of XP_002588697.1. The co-occurrence of a Lectin_C domain and fn2 domains, however is refuted by ESTs that match the region linking these two domain types. Tblastn searches of the database ESTs with XP_002588697.1 identified several ESTs that match the Lectin_C domain (ESTs BW716515, BW784251 andBW722680) or the fn2 region (BW803184) but not both parts of the chimeric XP_002588697.1 protein (**c**). The alignment shows only residues 241–480 of XP_002588697.1, containing the Lectin_C domain and the first two fn2 domains. Note that ESTs BW716515, BW784251, BW722680 match just the Lectin_C domain and define the stop codon of the upstream protein containing the Lectin_C domain. Also note that EST BW803184, that matches the fn2 domains, defines the N-terminal secretory signal peptide (residues underlined) of the downstream protein containing fn2 domain(s).

The second category (i.e. reciprocal best match failed to identify orthologs in Swiss-Prot) includes ortholog-pairs with identical but novel domain architectures (Supplementary Table S2) that are apparently unique to *Branchiostoma*, such as those containing Pfam-A domain 7tm_2 (017700_PFF0, 167280_PRF0, 18470_PRF0) or Pfam-A domain Neur_chan_memb (269420_PRF0). However, given the similarity of the two genomes and the similarity of gene-finding techniques, the identity of domain architecture of lancelet orthologs does not guarantee that they are valid Branchiostome-specific innovations. In some cases the innovation is an artefact: error convergence in genome annotation may yield similarly erroneous predictions. This danger may be illustrated with the case of protein 018300_PFF0 of *B. belcheri* and its *B. floridae* ortholog, XP_002588697.1 that contain a Lectin_C domain and five tandem fn2 domains. The co-occurrence of a Lectin_C domain and fn2 domains, however is refuted by ESTs that match the region linking these two domain types (Fig. 5). Tblastn searches of the database of *Branchiostoma* ESTs with the 018300_PFF0 and XP_002588697.1 proteins identified several ESTs (BW716515, BW784251, BW722680 and BW803184) that originate from this region but they argue against the fusion of the Lectin_C domain to the fn2 domains: they match either the Lectin_C domain (BW716515, BW784251, BW722680) or the fn2 region (BW803184) but not both parts of the chimeric XP_002588697.1 protein. ESTs BW716515, BW784251, BW722680 identified the stop codon of the upstream gene encoding the Lectin_C domain, whereas EST BW803184 defines the N-terminal secretory signal peptide of the protein containing fn2 domain(s) encoded by a downstream gene (Fig. 5).

## Conclusion

Cephalochordates are frequently referred to as ‘living fossils’ since they closely resemble fossil chordates from Cambrian strata. This extremely low level of morphological evolution is congruent with a low rate of evolution: cephalochordates were shown to be evolving more slowly than the slowest evolving vertebrate known, the elephant shark^37^.

In view of the low amino acid substitution rate and low level of morphological evolution in cephalochordates, it was surprising that the analysis of the proteomes of *B. belcheri* and *B. floridae* by Huang *et al.*^7^ suggested that they display the highest rates of domain combination acquisition known so far in metazoans. The authors hypothesized that the explanation for such an exceptional creativity is that lancelets retained high transposable element activity and an active exon-shuffling process.

This hypothesis, however, fails to provide an explanation for the apparent contradiction between the extreme morphological conservation of lancelets and the high rate of proteome innovation.

In our earlier work we have pointed out that creation of novel proteins are inextricably linked to major evolutionary innovations and increased organismic complexity such as those that accompanied the evolution of metazoa and vertebrates^13^,^38^, therefore it seemed to be surprising that major changes in the complexity of the lancelet proteomes are not reflected in major changes in their phenotype.

In the present work we provided evidence that the conclusion that the rate of protein innovation is exceptionally high in lancelets may be unjustified: the differences in domain architecture of orthologous proteins of various amphioxus species are more likely to reflect gene prediction errors than true innovation.

## Methods

We have used the genome data of Chinese lancelet (including annotations, proteins, transcripts; http://mosas.sysu.edu.cn/genome/download_data.php). One hundred proteins of *B. belcheri* were randomly selected from the dataset Branchiostoma.belcheri HapV2_proteins.fa and their equivalents were identified in other releases of predicted proteins (Branchiostoma.belcheri v15h11.r2_protein.fa and Branchiostoma.belcheri v18h27.r3_ref_protein.fa). Orthologs of the selected proteins were identified by the reciprocal best-hit method using NCBI’s non-redundant database of *B. floridae* proteins and the high quality, manually curated Swiss-Prot database.

The domain architectures of proteins (defined as the linear sequence of Pfam-A domains) were determined using Pfam (http://pfam.xfam.org)^32^. Since Huang *et al.*^7^ used only Pfam-A domain types in their analyses we have also restricted our analyses to Pfam-A domains.

Secretory signal peptides of proteins were predicted with SignalP 4.0 (http://www.cbs.dtu.dk/services/SignalP-4.0)^39^.

## Additional Information

**How to cite this article**: Bányai, L. and Patthy, L. Putative extremely high rate of proteome innovation in lancelets might be explained by high rate of gene prediction errors. *Sci. Rep.* **6**, 30700; doi: 10.1038/srep30700 (2016).

## Supplementary Material

Supplementary Information

## Figures and Tables

**Table 1:** Statistics of orthologous proteins of *B. belcheri* and *B. floridae.*

	R1	R2	R3		
Ortholog of the given *B. belcheri* protein					
is present in the *B. floridae* proteome	93	93	93		
is absent in the *B. floridae* proteome	7	7	7		
Ortholog-pairs of *B. belcheri* and in *B. floridae*					
have the same Domain Architecture	53	44	51		
have different Domain Architecture	40	49	42		
Ortholog-pairs of *B. belcheri* and in B. floridae*					
have the same Domain Architecture*	93	93	93		
have different Domain Architecture*	0	0	0		
are present in the Swiss-Prot database	74	74	74		
are absent in the Swiss-Prot database	23	23	23		
Orthologs in Swiss-Prot have Domain Architectures					
identical with those of					
both *B. belcheri* and *B. floridae*	29	23	30		
* B. belcheri* only	15	14	17		
* B. floridae* only	8	15	7		
neither *B. belcher*i nor *B. floridae*	22	22	18		

One hundred proteins of *B. belcheri*, containing at least two Pfam-A domains, were selected from dataset Branchiostoma.belcheri_HapV2_proteins.fa (release 1, R1) and their equivalents were identified in dataset Branchiostoma.belcheri_v15h11.r2_protein.fa (release 2, R2) and dataset Branchiostoma.belcheri_v18h27.r3_ref_protein.fa (release 3, R3). Orthologs of the selected proteins were identified by the reciprocal best-hit method, using the *B. floridae* section of NCBI’s non-redundant protein sequence database and UniProt KB’s Swiss-Prot database. The domain architectures of the orthologs (defined as the linear sequence of Pfam-A domains) were compared as described in the main text. The domain architectures of *B. belcheri* and *B. floridae* proteins are compared in Supplementary Tables 1 and 2, the domain architectures of the *B. belcheri* proteins from the three different releases are compared in Supplementary Table 3.

*The lines marked with an asterisk refer to analyses of protein sequences corrected by FixPred. The observation that FixPred correction eliminates domain architecture differences of orthologs of *B. belcheri* and *B. floridae* indicates that these differences were due to errors of gene prediction.
