# Supplementary material for: Putative extremely high rate of proteome innovation in lancelets might be explained by high rate of gene prediction errors
Source: Sci Rep. 2016 Aug 1;6:30700. doi: 10.1038/srep30700 (PMC4967905; doi:10.1038/srep30700)
Supplement: Supplementary Information [file srep30700-s1.pdf]

Supplementary File

Putative extremely high rate of proteome innovation in lancelets might be explained by high rate of gene prediction errors.

László Bányai and László Patthy

Institute of Enzymology, Research Centre for Natural Sciences, Hungarian Academy of Sciences, Budapest, P.O. Box 286, H-1519, Hungary,

Corresponding author: László Patthy,

POBox address: Budapest, P.O. Box 286, H-1519, Hungary,

Street address: Budapest, Magyar tudósok körútja 2, H-1117, Hungary

Tel: (361) 382 6751

Supplementary Table S1. Comparison of the domain architectures of orthologous proteins of *B. belcheri* and *B. floridae.* One hundred proteins of *B. belcheri*, containing at least two Pfam-A domains, were selected and their *B. floridae* orthologs (if any) were identified by the reciprocal best-hit method using a cut off value of 60% protein sequence identity. [1]Amino acid sequence identity over the aligned region. [2]Domain architecture is defined as the linear sequence of Pfam-A domains. Domains that differ in the domain architectures of orthologs from the two amphioxus species are highlighted in bold underline. The table compares the domain architectures of proteins that have orthologs in the high quality Swiss-Prot database. Entries whose domain architecture is identical with those of orthologs in the Swiss-Prot database are marked with an asterisk*.

**Sequence ID**<br>***B. belcheri***	 ***B. floridae***	**Protein sequence identity (%)[1]**	**Domain architecture[2]**<br>***B. belcheri***	 ***B. floridae***
001440_PFF0*	XP_002598316.1*	87	P4Ha_N<br>2OG-FeII_Oxy_3	P4Ha_N<br>2OG-FeII_Oxy_3
002260_PFF0*	XP_002598117.1*	86	Activin_recp<br>Pkinase_ Tyr	Activin_recp<br>Pkinase_ Tyr
003500_PFF0*	XP_002598733.1*	82	CLCA<br>VWA_2	CLCA<br>VWA_2
003850_PFF0*	XP_002597871.1	92	Pep_M12B_propep<br>Reprolysin_5<br><br>Disintegrin	Pep_M12B_propep<br>Reprolysin_5<br>**Pep_M12B_propep**<br>Disintegrin
012350_PFF0*	XP_002604729.1*	80	Kazal_2<br>SPARC_Ca_bdg<br>Thyroglobulin_1	Kazal_2<br>SPARC_Ca_bdg<br>Thyroglobulin_1
017590_PFF0*	XP_002598432.1*	77	Cu-oxidase_3<br>Cu-oxidase<br>Cu-oxidase_2	Cu-oxidase_3<br>Cu-oxidase<br>Cu-oxidase_2
052490_PFF0*	XP_002608045.1*	93	Fz<br>NTR	Fz<br>NTR
058270_PFF0	XP_002586055.1	72	I-set<br>Fz<br>Kringle	I-set<br>Fz<br>Kringle<br>**Fz**<br>**Kringle**<br>**I-set**<br>**Pkinase_Tyr**<br>**I-set**<br>**Pkinase_Tyr**
078830_PFF0*	XP_002596432.1*	93	CPSase_L_chain<br>CPSase_L_D2<br>Biotin_carb_C<br>HMGL-like<br>PYC_OADA<br>Biotin_lipoyl	CPSase_L_chain<br>CPSase_L_D2<br>Biotin_carb_C<br>HMGL-like<br>PYC_OADA<br>Biotin_lipoyl
080250_PFF0	XP_002598506.1	94	**LRR_8**<br>**I-set**<br>Wnt	 <br>Wnt

080250_PFF0	XP_002598508.1	94	**LRR_8**	
			**I-set**	
			Wnt	Wnt
091850_PFF0	XP_002591393.1	75	Gal_Lectin	Gal_Lectin
			OLF	OLF
			HRM	HRM
			**GPS**	
			GAIN	GAIN
			7tm_2	7tm_2
102470_PFF0*	XP_002608336.1	98	RNA_pol_Rpb2_1	RNA_pol_Rpb2_1
			RNA_pol_Rpb2_2	RNA_pol_Rpb2_2
			RNA_pol_Rpb2_3	RNA_pol_Rpb2_3
			RNA_pol_Rpb2_4	RNA_pol_Rpb2_4
			RNA_pol_Rpb2_5	RNA_pol_Rpb2_5
			RNA_pol_Rpb2_6	RNA_pol_Rpb2_6
			**RNA_pol_Rpb2_7**	
103400_PFF0	XP_002593834.1*	91	**Ank_3**	
			**Ank_2**	
			**Ank_4**	
			Ank_2	Ank_2
			Arm	Arm
			**Arm**	
114280_PFF0	XP_002608942.1	89	C2	C2
			C2	C2
			FerI	FerI
			C2	C2
			FerB	FerB
			C2	C2
			C2	C2
			C2	C2
			C2	C2
			Ferlin_C	Ferlin_C
117350_PFF0*	XP_002599461.1*	89	TGFb_propeptide	TGFb_propeptide
			TGF_beta	TGF_beta
118940_PFF0*	XP_002587581.1*	86	HAD_2	HAD_2
			APH	APH
			Acyl-CoA_dh_N	Acyl-CoA_dh_N
			Acyl-CoA_dh_M	Acyl-CoA_dh_M
			Acyl-CoA_dh_1	Acyl-CoA_dh_1
123650_PFF0*	XP_002603715.1	95	CAP_GLY	CAP_GLY
			**Dynactin**	
123650_PFF0*	XP_002603716.1	93	**CAP_GLY**	
			Dynactin	Dynactin
127590_PFF0	XP_002614044.1*	86	Kazal_2	Kazal_2
			Kazal_2	Kazal_2
			Kazal_2	Kazal_2
			SEA	SEA
			Kazal_2	Kazal_2
			Kazal_2	Kazal_2
			Kazal_2	Kazal_2
			Kazal_2	Kazal_1
			Kazal_1	Kazal_1

			Laminin_EGF	Laminin_EGF
			Laminin_EGF	Laminin_EGF
				**Kazal_2**
				**EGF**
				**Laminin_G_1**
				**Laminin_G_1**
				**EGF**
				**Laminin_G_1**
127600_PFF0	XP_002614044.1*	90		**Kazal_2**
				**Kazal_2**
				**Kazal_2**
				**SEA**
				**Kazal_2**
				**Kazal_2**
				**Kazal_2**
				**Kazal_1**
				**Kazal_1**
				**Laminin_EGF**
				**Laminin_EGF**
			Kazal_2	Kazal_2
			EGF	EGF
			Laminin_G_1	Laminin_G_1
			Laminin_G_1	Laminin_G_1
			EGF	EGF
			Laminin_G_1	Laminin_G_1
128050_PFF0	XP_002603923.1	71	TSP_1	TSP_1
				**TSP_1**
			Ldl_recept_a	Ldl_recept_a
			MACPF	MACPF
			TSP_1	TSP_1
			GCC2_GCC3	GCC2_GCC3
133320_PFF0	XP_002610402.1	91	SRCR	SRCR
			Kringle	Kringle
			SRCR	SRCR
			Trypsin	Trypsin
135460_PFF0*	XP_002605157.1*	85	Fz	Fz
			Frizzled	Frizzled
144220_PFF0	XP_002591459.1	92	RICTOR_N	RICTOR_N
			RICTOR_M	RICTOR_M
			RasGEF_N_2	RasGEF_N_2
			RICTOR_V	RICTOR_V
152200_PFF0*	–		FHA	
			CEP170_C	
153460_PFF0*	–		Ank_2	
			Ank_2	
			Ank_2	
			Ion_trans	
157010_PFF0*	XP_002611039.1	87	EF-hand_7	EF-hand_7
			Ferric_reduct	Ferric_reduct
			**FAD_binding_8**	
			NAD_binding_6	NAD_binding_6

157070_PFF0*	XP_002611055.1*	95	ANAPC10<br>HECT	ANAPC10<br>HECT
157720_PFF0	XP_002612291.1	90	 HYR<br>GCC2_GCC3	**Troponin**<br>HYR<br>GCC2_GCC3
158710_PFF0	XP_002600118.1	95	tRNA-synt_1<br>Anticodon_1<br>Val_tRNA-synt_C	tRNA-synt_1<br>Anticodon_1<br>Val_tRNA-synt_C
158830_PFF0*	XP_002600149.1*	83	IGFBP<br>VWC<br>TSP_1	IGFBP<br>VWC<br>TSP_1
160400_PFF0	XP_002601973.1	93	Integrin_beta<br>Integrin_B_tail<br>Integrin_b_cyt	Integrin_beta<br>Integrin_B_tail<br>Integrin_b_cyt
161590_PFF0	XP_002592408.1	98	**FERM_M**<br>**IRS**<br>**Talin_middle**<br>**I_LWEQ**<br>**VBS**<br>VBS<br>I_LWEQ	 <br><br><br><br>VBS<br>I_LWEQ
161590_PFF0	XP_002592411.1	92	FERM_M<br>IRS<br>Talin_middle<br>**I_LWEQ**<br>**VBS**<br>**VBS**<br>**I_LWEQ**	FERM_M<br>IRS<br>Talin_middle
161590_PFF0	XP_002592410.1	92	**FERM_M**<br>**IRS**<br>**Talin_middle**<br>I_LWEQ<br>VBS<br>**VBS**<br>**I_LWEQ**	 <br><br>I_LWEQ<br>VBS
172520_PRF0*	XP_002611318.1*	97	Fz<br>NTR	Fz<br>NTR
185920_PRF0	XP_002606276.1*	92	An_peroxidase<br><br>EGF_CA	An_peroxidase<br>**Sushi**<br>EGF_CA
186040_PRF0*	XP_002606276.1*	91	An_peroxidase<br>Sushi<br>EGF_CA	An_peroxidase<br>Sushi<br>EGF_CA
189350_PRF0*	XP_002596820.1	99	FbpA<br>**DUF814**<br>**DUF3441**	FbpA

189350_PRF0*	XP_002596821.1	80	**FbpA**	
			DUF814	DUF814
			DUF3441	DUF3441
192660_PRF0*	XP_002586029*	95	ThiF	ThiF
			E1_FCCH	E1_FCCH
			E1_4HB	E1_4HB
			ThiF	ThiF
			UBA_e1_thiolCys	UBA_e1_thiolCys
			E1_UFD	E1_UFD
196330_PRF0	XP_002594642.1*	86	Ion_trans	Ion_trans
			Ion_trans	Ion_trans
			**Ion_trans**	
			**Ion_trans**	
207610_PRF0*	XP_002610947.1*	88	TGFb_propeptide	TGFb_propeptide
			TGF_beta	TGF_beta
208940_PRF0*	XP_002607923.1	95	**ANF_receptor**	
			Pkinase_Tyr	Pkinase_Tyr
			Guanylate_cyc	Guanylate_cyc
209230_PRF0	XP_002610589.1*	88	Neur_chan_LBD	Neur_chan_LBD
			Neur_chan_memb	Neur_chan_memb
			**Neur_chan_LBD**	
			**Neur_chan_memb**	
211270_PRF0*	XP_002591619.1*	88	BAR_3	BAR_3
			PH	PH
			ArfGap	ArfGap
			Ank_2	Ank_2
215230_PRF0*	XP_002589217.1*	88	PMT	PMT
			MIR	MIR
			PMT_4TMC	PMT_4TMC
215230_PRF0*	XP_002589229.1*	90	PMT	PMT
			MIR	MIR
			PMT_4TMC	PMT_4TMC
222490_PRF0*	XP_002598311.1	97	**DMAP_binding**	
			DNMT1-RFD	DNMT1-RFD
			zf-CXXC	zf-CXXC
			BAH	BAH
			BAH	BAH
			DNA_methylase	DNA_methylase
223320_PRF0	XP_002601301.1	79	Pep_M12B_propep	Pep_M12B_propep
			Reprolysin	Reprolysin
			TSP_1	TSP_1
			ADAM_spacer1	ADAM_spacer1
			TSP_1	TSP_1
			TSP_1	TSP_1
			TSP_1	TSP_1
			TSP_1	TSP_1
				**TSP_1**
				**TSP_1**
				**TSP_1**
227800_PRF0	XP_002608349.1	63	SRCR	SRCR

			SRCR	SRCR
			SRCR	SRCR
			Lysyl_oxidase	Lysyl_oxidase
				**<u>SRCR</u>**
				**<u>Lysyl_oxidase</u>**
				**<u>Lysyl_oxidase</u>**
237250_PRF0	XP_002612866.1	91		**<u>ANATO</u>**
				**<u>A2M</u>**
				**<u>Thiol-ester_cl</u>**
				**<u>A2M_comp</u>**
			A2M_recep	A2M_recep
			NTR	NTR
237260_PRF0	XP_002612866.1	90	**A2M_N**	
			**A2M_N_2**	
			ANATO	ANATO
			A2M	A2M
			Thiol-ester_cl	Thiol-ester_cl
			A2M_comp	A2M_comp
				**A2M_recep**
				**NTR**
237260_PRF0	AF391289_5	92	A2M_N	A2M_N
			A2M_N_2	A2M_N_2
			ANATO	ANATO
			A2M	A2M
			**<u>Thiol-ester_cl</u>**	
			**<u>A2M_comp</u>**	
246010_PRF0*	XP_002613969.1*	72	PG_binding_1	PG_binding_1
			Peptidase_M10	Peptidase_M10
			Hemopexin	Hemopexin
			Hemopexin	Hemopexin
			Hemopexin	Hemopexin
			Hemopexin	Hemopexin
247370_PRF0*	–		WIF	
			Pkinase_Tyr	
248080_PRF0	XP_002591824.1*	92	**<u>EGF_3</u>**	
			Sushi	Sushi
			Sushi	Sushi
			VWA	VWA
			Trypsin	Trypsin
253950_PRF0*	XP_002610496.1*	97	Histone	Histone
			RhoGEF	RhoGEF
			PH	PH
			RasGEF_N	RasGEF_N
			RasGEF	RasGEF
253960_PRF0*	XP_002610495.1*	96	zf-Sec23_Sec24	zf-Sec23_Sec24
			Sec23_trunk	Sec23_trunk
			Sec23_BS	Sec23_BS
			Sec23_helical	Sec23_helical
			Gelsolin	Gelsolin
254170_PRF0*	XP_002595526.1*	87	ABC_membrane	ABC_membrane
			ABC_tran	ABC_tran
			ABC_membrane	ABC_membrane

			ABC_tran	ABC_tran
256530_PRF0	B6MUN4.1	64	**Myb_DNA-binding**	
			SWIRM	SWIRM
				**JAB**
256520_PRF0	B6MUN4.1	76		**SWIRM**
			JAB	JAB
257610_PRF0*	XP_002587133.1*	84	EST1	EST1
			EST1_DNA_bind	EST1_DNA_bind
			EST1_DNA_bind	EST1_DNA_bind
			PIN_4	PIN_4
258810_PRF0	XP_002605408.1*	60	Glyco_transf_29	Glyco_transf_29
			**Glyco_transf_29**	
258870_PRF0*	XP_002595259.1	98	**STAT_int**	
			STAT_alpha	STAT_alpha
			STAT_bind	STAT_bind
			SH2	SH2
258870_PRF0*	XP_002585719.1	92	STAT_int	STAT_int
			**STAT_alpha**	
			**STAT_bind**	
			**SH2**	
258870_PRF0*	XP_002595260.1	100	**STAT_int**	
			**STAT_alpha**	
			STAT_bind	STAT_bind
			**SH2**	
258870_PRF0*	XP_002595256.1	96	**STAT_int**	
			**STAT_alpha**	
			STAT_bind	STAT_bind
			SH2	SH2
259020_PRF0*	XP_002600999.1*	97	GRAM	GRAM
			GRAM	GRAM
			RabGAP-TBC	RabGAP-TBC
272770_PRF0*	XP_002601715.1*	90	AAA_11	AAA_11
			AAA_11	AAA_11
			AAA_12	AAA_12
283240_PRF0	CAA06854.1	95	Reeler	Reeler
			Spond_N	Spond_N
			fn3	fn3
			TSP_1	TSP_1
			fn3	fn3
			TSP_1	TSP_1
			TSP_1	TSP_1
293630_PRF0*	XP_002613483.1*	92	Chorein_N	Chorein_N
			ATG_C	ATG_C
293740_PRF0	XP_002613924.1	92		**VPS13**
				**VPS13_mid_rpt**
				**UBA**
			**SHR-BD**	**SHR-BD**

			**VPS13_C**	**VPS13_C**
209920_PRM0	XP_002613924.1	89		**VPS13**
				**VPS13_mid_rpt**
			UBA	UBA
				**SHR-BD**
				**VPS13_C**
209930_PRF0	XP_002613924.1	81		**VPS13**
				**VPS13_mid_rpt**
				**UBA**
				**SHR-BD**
				**VPS13_C**
209940_PRF0	XP_002613924.1	86	**Chorein_N**	
			VPS13	VPS13
			VPS13_mid_rpt	VPS13_mid_rpt
				**UBA**
				**SHR-BD**
				**VPS13_C**
209940_PRF0	XP_002613923.1	94	Chorein_N	Chorein_N
			**VPS13**	
			**VPS13_mid_rpt**	
321850_PRF0*	XP_002585883.1*	85	ANF_receptor	ANF_receptor
			Lig_chan-Glu_bd	Lig_chan-Glu_bd
			Lig_chan	Lig_chan
319230_PRF0*	XP_002600703.1	77	cNMP_binding	cNMP_binding
			DEP	DEP
			cNMP_binding	cNMP_binding
			**RasGEF_N**	
			**RasGEF**	
319230_PRF0*	XP_002600702.1	95	**cNMP_binding**	
			**DEP**	
			**cNMP_binding**	
			**RasGEF_N**	
			RasGEF	RasGEF
325460_PRF0	XP_002608790.1*	88	FG-GAP	FG-GAP
			FG-GAP	FG-GAP
			Integrin_alpha2	Integrin_alpha2
				Integrin_alpha
320370_PRF0	XP_002594515.1	72	**zf-RING_5**	
				**zf-C3HC4_3**
			zf-B_box	zf-B_box
			fn3	fn3
			**SPRY**	
325780_PRF0*	XP_002602117.1 91*	91	Glycos_transf_2	Glycos_transf_2
			Ricin_B_lectin	Ricin_B_lectin
328450_PRF0	XP_002607180.1*	79		**WAP**
				**Kazal_2**
				**Kunitz_BPTI**
			Kunitz_BPTI	Kunitz_BPTI
			NTR	NTR

328460_PRF0	XP_002607180.1*	85	WAP	WAP
			Kazal_2	Kazal_2
			Kunitz_BPTI	Kunitz_BPTI
			Kunitz_BPTI	Kunitz_BPTI
				**NTR**
328470_PRF0	XP_002607185.1	96	ABC_membrane	ABC_membrane
			ABC_tran	ABC_tran
			ABC_membrane	ABC_membrane
			ABC_tran	ABC_membrane
			ABC_membrane	ABC_membrane
			ABC_tran	ABC_membrane
			ABC_membrane	ABC_membrane
			ABC_tran	ABC_tran
328810_PRF0	XP_002592166.1	91	zf-HC5HC2H	zf-HC5HC2H
			FYRN	FYRN
			FYRC	FYRC
			SET	SET
328940_PRF0	XP_002612925.1	90	**dsrm**	
			**dsrm**	
			A_deamin	A_deamin
			**A_deamin**	
328940_PRF0	XP_002612924.1	99	dsrm	dsrm
			dsrm	dsrm
			**A_deamin**	
			**A_deamin**	
329040_PRF0*	XP_002612907.1*	82	Flavodoxin_1	Flavodoxin_1
			FAD_binding_1	FAD_binding_1
			NAD_binding_1	NAD_binding_1
329370_PRF0*	XP_002611195.1	70		**Catalase**
			PLAT	PLAT
			Lipoxygenase	Lipoxygenase
329970_PRF0	XP_002606233.1	92	DAG_kinase_N	DAG_kinase_N
			C1_1	C1_1
			C1_1	C1_1
			DAGK_cat	DAGK_cat
			DAGK_acc	DAGK_acc
330040_PRF0*	–		Activin_recp	
			Pkinase	
330210_PRF0*	XP_002602990.1	98	EF-hand	EF-hand
			Mito_carr	Mito_carr
			Mito_carr	Mito_carr
			Mito_carr	Mito_carr
				**Peptidase M2**

Supplementary Table S2. Comparison of the domain architectures of orthologous proteins of *B. belcheri* and *B. floridae.* One hundred proteins of *B. belcheri*, containing at least two Pfam-A domains, were selected and their *B. floridae* orthologs (if any) were identified by the reciprocal best-hit method using a cut off value of 60% protein sequence identity. [1]Amino acid sequence identity over the aligned region. [2]Domain architecture is defined as the linear sequence of Pfam-A domains. Domains that differ in the domain architectures of orthologs from the two amphioxus species are highlighted in bold underline. The table compares the domain architectures of proteins that have no orthologs in the high quality Swiss-Prot database.

**Sequence ID**		**Protein sequence identity (%)[1]**	**Domain architecture[2]**	
B. belcheri	***B. floridae***		***B. belcheri***	***B. floridae***
000200_PFF0	AAN62850.1	85	V-set<br>CBM_14	V-set<br>CBM_14
001280_PFF0	XP_002608265.1	88	CBM_14<br>ILEI	CBM_14<br>ILEI
017700_PFF0	XP_002598460.1	85	Gal_Lectin<br>Gal_Lectin<br>GPS<br>7tm_2	Gal_Lectin<br>Gal_Lectin<br>GPS<br>7tm_2
018300_PFF0	XP_002588697.1	68	Lectin_C<br>fn2<br>fn2<br>fn2<br>fn2<br>fn2	Lectin_C<br>fn2<br>fn2<br>fn2<br>fn2<br>fn2
040970_PFF0	XP_002596007.1	95	kringle<br>kringle<br>trypsin	kringle<br>kringle<br>trypsin
068220_PFF0	XP_002586877.1	79	fn2<br>fn2<br>fn2<br>fn2<br>CUB<br>CUB	fn2<br>fn2<br>fn2<br>fn2<br>CUB<br>CUB
080050_PFF0	XP_002606926.1	92	N-glycanase_N<br>N-glycanase_C	N-glycanase_N<br>N-glycanase_C
124390_PFF0	–		cEGF<br>FXa_inhibition<br>EGF_CA<br>EGF_CA<br>NIDO<br>EGF_CA<br>EGF_CA<br>SEA<br>EGF_CA	
159900_PFF0	XP_002600642.1	82	AAA_8<br>MT<br>AAA_9<br><br>Dynein_heavy	AAA_8<br>MT<br>AAA_9<br>AAA_9<br>Dynein_heavy

167280_PRF0	XP_002611740.1	66		Lectin_C
			Kringle	Kringle
			Somatomedin_B	Somatomedin_B
			7tm_2	7tm_2
173360_PRF0	XP_002593467.1	92	Astacin	Astacin
			kringle	kringle
180420_PRF0	XP_002609978.1	90		**5-FTHF_cyc-lig**
			Somatomedin_B	Somatomedin_B
			7tm_2	7tm_2
184730_PRF0	XP_002597992.1	65	Ldl_recept_a	Ldl_recept_a
			Kringle	Kringle
			Kringle	Kringle
			7tm_2	7tm_2
188610_PRF0	XP_002609579.1	86	ASC	ASC
			F5_F8_type_C	F5_F8_type_C
			F5_F8_type_C	F5_F8_type_C
			Kringle	Kringle
			ASC	ASC
196740_PRF0	XP_002601342.1	89	**Fibrinogen_C**	
			**Fibrinogen_C**	
			**I-set**	
			**fn3**	
			**fn3**	
			**fn3**	
			**fn3**	
			Y_phosphatase	Y_phosphatase
			Y_phosphatase	Y_phosphatase
196740_PRF0	XP_002601345.1	78	Fibrinogen_C	Fibrinogen_C
			Fibrinogen_C	Fibrinogen_C
			**I-set**	
			fn3	fn3
			**fn3**	
			**fn3**	
			**fn3**	
			**Y_phosphatase**	
			**Y_phosphatase**	
200810_PRF0	XP_002608508.1	75	Astacin	Astacin
			CUB	CUB
			MAM	MAM
258300_PRF0	XP_002605710.1	90	**Ldl_recept_a**	
			**SRCR**	
			**Ldl_recept_a**	
			Trypsin	Trypsin
258300_PRF0	XP_002605711.1	90	**Ldl_recept_a**	
			SRCR	SRCR
			**Ldl_recept_a**	
			**Trypsin**	
260260_PRF0	–		BACK	
			Kelch_1	
269420_PRF0	XP_002605854.1	73	CUB	CUB

			Ldl_recept_a<br>Fz<br>Kringle<br>Neur_chan_memb	Ldl_recept_a<br>Fz<br>Kringle<br>Neur_chan_memb
304470_PRF0	XP_002587583.1	88	Ldl_recept_a<br>VWA<br>Zona_pellucida	Ldl_recept_a<br>VWA<br>Zona_pellucida
320550_PRF0	–		Kringle<br>Kringle<br>Lectin_C<br>CUB<br>Lectin_C<br>MAM<br>Lectin_C<br>Kringle<br>SRCR	
329690_PRF0	XP_002611153.1	90	BTB<br>BTB<br>BACK<br>Kelch_1<br>Kelch_1<br>Kelch_1<br>Kelch_1	BTB<br>BTB<br>BACK<br>Kelch_1<br>Kelch_1<br>Kelch_1<br>Kelch_1
329890_PRF0	XP_002606025.1	77	COR<br>Death	COR<br>Death

Supplementary Table S3. Comparison of the domain architectures of proteins of *B. belcheri* selected from dataset Branchiostoma.belcheri_HapV2_proteins.fa (release 1) with those of their equivalents in the dataset Branchiostoma.belcheri_v15h11.r2_protein.fa (release 2) and dataset Branchiostoma.belcheri_v18h27.r3_ref_protein.fa (release 3). One hundred proteins of *B. belcheri*, containing at least two Pfam-A domains, were selected from release 1 and their matches were identified in release 2 and release 3. [2]Domain architecture (DA) is defined as the linear sequence of Pfam-A domains. Entries whose domain architecture is identical with those of orthologs in the high quality Swiss-Prot database are marked with an asterisk*. Mispredicted entries with DA that deviates from those of orthologous Swiss-Prot entries are highlighted in bold underline. The data are arranged in two categories based on the presence or absence of orthologs in the Swiss-Prot database.

Sequence ID release 1	DA	Sequence ID release 2	DA	Sequence ID release 3	DA
**Orthologs are present in the Swiss-Prot database**					
001440_PFF0*	P4Ha_N<br>2OG-FeII_Oxy_3	**053450F.t1**	P4Ha_N	089550F.t1*	P4Ha_N<br>2OG-FeII_Oxy_3
002260_PFF0*	Activin_recp<br>Pkinase_ Tyr	243600F.t1*	Activin_recp<br>Pkinase_ Tyr	297980F.t2*	Activin_recp<br>Pkinase_ Tyr
003500_PFF0*	CLCA<br>VWA_2	041250R.t1*	CLCA<br>VWA_2	265240R.t1*	CLCA<br>VWA_2
003850_PFF0*	Pep_M12B_propep<br>Reprolysin_5<br>Disintegrin	**062250R.t1**	Reprolysin_5<br>Disintegrin	095570F.t1*	Pep_M12B_propep<br>Reprolysin_5<br>Disintegrin
012350_PFF0*	Kazal_2<br>SPARC_Ca_bdg<br>Thyroglobulin_1	229830F.t1*	Kazal_2<br>SPARC_Ca_bdg<br>Thyroglobulin_1	197610F.t1*	Kazal_2<br>SPARC_Ca_bdg<br>Thyroglobulin_1
017590_PFF0*	Cu-oxidase_3<br>Cu-oxidase<br>Cu-oxidase_2	002580R.t1*	Cu-oxidase_3<br>Cu-oxidase<br>Cu-oxidase_2	**204890R.t2**	Cu-oxidase_3<br>Cu-oxidase<br>Cu-oxidase_2<br>Cu-oxidase
052490_PFF0*	Fz<br>NTR	017500F.t1*	Fz<br>NTR	127920R.t1*	Fz<br>NTR
**058270_PFF0**	I-set<br>Fz<br>Kringle	142670R.t2	I-set<br>Fz<br>Kringle<br>I-set<br>Pkinase_Tyr	192300R.t2	I-set<br>Fz<br>Kringle<br>I-set<br>Pkinase_Tyr
078830_PFF0*	CPSase_L_chain<br>CPSase_L_D2<br>Biotin_carb_C<br>HMGL-like<br>PYC_OADA<br>Biotin_lipoyl	097650F.t2*	CPSase_L_chain<br>CPSase_L_D2<br>Biotin_carb_C<br>HMGL-like<br>PYC_OADA<br>Biotin_lipoyl	252380R.t2*	CPSase_L_chain<br>CPSase_L_D2<br>Biotin_carb_C<br>HMGL-like<br>PYC_OADA<br>Biotin_lipoyl
**080250_PFF0**	LRR_8<br>I-set<br>Wnt	**001910R.t1**	Wnt	062470F.t1*	Wnt

			Wnt		
091850_PFF0	Gal_Lectin	**324940R.t1**	Gal_Lectin	191840R.t2	Gal_Lectin
	OLF		OLF		OLF
	HRM		HRM		HRM
	GAIN		GAIN		GAIN
			GAIN		
	GPS		GPS		GPS
	7tm_2		7tm_2		7tm_2
102470_PFF0*	RNA_pol_Rpb2_1	010690R.t1*	A_pol_Rpb2_1	282790F.t1*	A_pol_Rpb2_1
	RNA_pol_Rpb2_2		A_pol_Rpb2_2		A_pol_Rpb2_2
	RNA_pol_Rpb2_3		A_pol_Rpb2_3		A_pol_Rpb2_3
	RNA_pol_Rpb2_4		A_pol_Rpb2_4		A_pol_Rpb2_4
	RNA_pol_Rpb2_5		A_pol_Rpb2_5		A_pol_Rpb2_5
	RNA_pol_Rpb2_6		A_pol_Rpb2_6		A_pol_Rpb2_6
	RNA_pol_Rpb2_7		A_pol_Rpb2_7		A_pol_Rpb2_7
**103400_PFF0**	Ank_3	**028040F.t1**		**043790F.t1**	
	Ank_2				
	Ank_4		Ank_4		Ank_4
			Ank_2		Ank_2
	Arm		Arm		Arm
	Arm		Arm		Arm
**114280_PFF0**	C2	261060R.t1*	C2	167750F.t1*	C2
	C2		C2		C2
	FerI		FerI		FerI
	C2		C2		C2
			FerA		FerA
	FerB		FerB		FerB
	C2		C2		C2
	C2		C2		C2
	C2		C2		C2
	C2		C2		C2
	Ferlin_C		Ferlin_C		Ferlin_C
117350_PFF0*	TGFb_propeptide	099520R.t1*	TGFb_propeptide	195530R.t1*	TGFb_propeptide
	TGF_beta		TGF_beta		TGF_beta
118940_PFF0*	HAD_2	092840R.t1*	HAD_2	003000F.t1*	HAD_2
	APH		APH		APH
	Acyl-CoA_dh_N		Acyl-CoA_dh_N		Acyl-CoA_dh_N
	Acyl-CoA_dh_M		Acyl-CoA_dh_M		Acyl-CoA_dh_M
	Acyl-CoA_dh_1		Acyl-CoA_dh_1		Acyl-CoA_dh_1
123650_PFF0*	CAP_GLY	265490R.t1*	CAP_GLY	121780F.t5*	CAP_GLY
	Dynactin		Dynactin		Dynactin
**127590_PFF0**		**303040R.t1**	Kazal_2	212090F.t4*	
			Kazal_2		
			Kazal_2		
			SEA		
			Kazal_2		
			Kazal_2		
			Kazal_2		
			Kazal_2		
			Kazal_1		
	Kazal_2		Kazal_2		Kazal_2
	Kazal_2		Kazal_2		Kazal_2
	Kazal_2		Kazal_2		Kazal_2
	SEA				SEA

	Kazal_2		Kazal_2		Kazal_2
	Kazal_2		Kazal_2		Kazal_2
	Kazal_2		Kazal_2		Kazal_2
	Kazal_1		Kazal_1		Kazal_1
	Kazal_1		Kazal_1		Kazal_1
	Laminin_EGF		Laminin_EGF		Laminin_EGF
	Laminin_EGF		Laminin_EGF		Laminin_EGF
			Kazal_2		Kazal_2
			EGF		EGF
			Laminin_G_1		Laminin_G_1
			Laminin_G_1		Laminin_G_1
			EGF		EGF
			Laminin_G_1		Laminin_G_1
**127600_PFF0**		**303040R.t1**	Kazal_2	212090F.t4*	
			Kazal_2		
			Kazal_2		
			SEA		
			Kazal_2		
			Kazal_2		
			Kazal_2		
			Kazal_2		
			Kazal_1		
			Kazal_2		Kazal_2
			Kazal_2		Kazal_2
			Kazal_2		Kazal_2
			SEA		SEA
			Kazal_2		Kazal_2
			Kazal_2		Kazal_2
			Kazal_2		Kazal_2
			Kazal_1		Kazal_1
			Kazal_1		Kazal_1
			Laminin_EGF		Laminin_EGF
			Laminin_EGF		Laminin_EGF
	Kazal_2		Kazal_2		Kazal_2
	EGF		EGF		EGF
	Laminin_G_1		Laminin_G_1		Laminin_G_1
	Laminin_G_1		Laminin_G_1		Laminin_G_1
	EGF		EGF		EGF
	Laminin_G_1		Laminin_G_1		Laminin_G_1
128050_PFF0	TSP_1	013570R.t2	TSP_1	174680F.t1	TSP_1
			TSP_1		TSP_1
	Ldl_recept_a		Ldl_recept_a		Ldl_recept_a
	MACPF		MACPF		MACPF
	TSP_1		TSP_1		TSP_1
	GCC2_GCC3		GCC2_GCC3		GCC2_GCC3
133320_PFF0	SRCR	250080R.t1	SRCR	228670R.t1	SRCR
	Kringle		Kringle		Kringle
	SRCR		SRCR		SRCR
	Trypsin		Trypsin		Trypsin
135460_PFF0*	Fz	222910F.t1*	Fz	**019690R.t1**	
	Frizzled		Frizzled		Frizzled
**144220_PFF0**		**045000R.t1**	RCC1	**081090R.t3**	RCC1
			RCC1		RCC1
			RCC1		RCC1
			RCC1		RCC1
	RICTOR_N		RICTOR_N		RICTOR_N
	RICTOR_M		RICTOR_M		RICTOR_M

	RasGEF_N_2		RasGEF_N_2		RasGEF_N_2
	RICTOR_V		RICTOR_V		RICTOR_V
152200_PFF0*	FHA	**098310R.t1**		251720F.t1*	FHA
	CEP170_C		CEP170_C		CEP170_C
153460_PFF0*	Ank_2	227850R.t1*	Ank_2	004550F.t1*	Ank_2
	Ank_2		Ank_2		Ank_2
	Ank_2		Ank_2		Ank_2
	Ion_trans		Ion_trans		Ion_trans
157010_PFF0*	EF-hand_7	030190R.t2*	EF-hand_7	276360F.t1*	EF-hand_7
	Ferric_reduct		Ferric_reduct		Ferric_reduct
	FAD_binding_8		FAD_binding_8		FAD_binding_8
	NAD_binding_6		NAD_binding_6		NAD_binding_6
157070_PFF0*	ANAPC10	**030090R.t1**	ANAPC10	276460F.t1*	ANAPC10
	HECT		HECT		HECT
			ANAPC10		
			HECT		
157720_PFF0		124730F.t1		**261920R.t2**	Troponin
	HYR		HYR		HYR
	GCC2_GCC3		GCC2_GCC3		GCC2_GCC3
**158710_PFF0**	tRNA-synt_1	**189810F.t1**	tRNA-synt_1	**259960R.t1**	tRNA-synt_1
	Anticodon_1		Anticodon_1		Anticodon_1
	Val_tRNA-synt_C				
158830_PFF0*	IGFBP	190070F.t1*	IGFBP	259710R.t1*	IGFBP
	VWC		VWC		VWC
	TSP_1		TSP_1		TSP_1
160400_PFF0	Integrin_beta	100620R.t1	Integrin_beta	196660R.t1	Integrin_beta
	Integrin_B_tail		Integrin_B_tail		Integrin_B_tail
	Integrin_b_cyt		Integrin_b_cyt		Integrin_b_cyt
**161590_PFF0**	FERM_M	**181770F.t1**	FERM_M	**085620R.t1**	FERM_M
	IRS				IRS
	Talin_middle		Talin_middle		Talin_middle
	I_LWEQ		I_LWEQ I_		LWEQ
	VBS		VBS		
	VBS		VBS		
	I_LWEQ				
**161590_PFF0**	FERM_M	**181780F.t1**		**085620R.t3**	
	IRS				
	Talin_middle				
	I_LWEQ				
	VBS				VBS
	VBS				VBS
	I_LWEQ		I_LWEQ		
172520_PRF0*	Fz	078910F.t1*	Fz	190570F.t1*	Fz
	NTR		NTR		NTR
**185920_PRF0**	An_peroxidase	**262810R.t1**	An_peroxidase	222960R.t1*	An_peroxidase
					Sushi
	EGF_CA		EGF_CA		EGF_CA

186040_PRF0*		**262130R.t2**	Peptidase_S10	222960R.t2*	
	An_peroxidase		An_peroxidase		An_peroxidase
	Sushi				Sushi
	EGF_CA		EGF_CA		EGF_CA
189350_PRF0*	FbpA	068710R.t1*	FbpA	238700F.t1*	FbpA
	DUF814		DUF814		DUF814
	DUF3441		DUF3441		DUF3441
192660_PRF0*	ThiF	079900F.t1*	ThiF	188840R.t1*	ThiF
	E1_FCCH		E1_FCCH		E1_FCCH
	E1_4HB		E1_4HB		E1_4HB
	ThiF		ThiF		ThiF
	UBA_e1_thiolCys		UBA_e1_thiolCys		UBA_e1_thiolCys
	E1_UFD		E1_UFD		E1_UFD
**196330_PRF0**	Ion_trans	038350R.t1*	Ion_trans	225110R.t1*	Ion_trans
	Ion_trans		Ion_trans		Ion_trans
	Ion_trans				
	Ion_trans				
207610_PRF0*	TGFb_propeptide	236010R.t1*	TGFb_propeptide	077270F.t1*	TGFb_propeptide
	TGF_beta		TGF_beta		TGF_beta
208940_PRF0*	ANF_receptor	067530R.t1*	ANF_receptor	281460F.t3*	ANF_receptor
	Pkinase_Tyr		Pkinase_Tyr		Pkinase_Tyr
	Guanylate_cyc		Guanylate_cyc		Guanylate_cyc
**209230_PRF0**	Neur_chan_LBD	**039070F.t1**	Neur_chan_LBD	**294360F.t1**	Neur_chan_LBD
	Neur_chan_memb		Neur_chan_memb		Neur_chan_memb
	Neur_chan_LBD		Neur_chan_LBD		Neur_chan_LBD
	Neur_chan_memb		Neur_chan_memb		Neur_chan_memb
211270_PRF0*	BAR_3	150370F.t1*	BAR_3	140660F.t1*	BAR_3
	PH		PH		PH
	ArfGap		ArfGap		ArfGap
	Ank_2		Ank_2		Ank_2
215230_PRF0*	PMT	311700R.t1*	PMT	296530R.t1*	PMT
	MIR		MIR		MIR
	PMT_4TMC		PMT_4TMC		PMT_4TMC
222490_PRF0*	DMAP_binding	053410R.t1*	DMAP_binding	089500R.t1*	DMAP_binding
	DNMT1-RFD		DNMT1-RFD		DNMT1-RFD
	zf-CXXC		zf-CXXC		zf-CXXC
	BAH		BAH		BAH
	BAH		BAH		BAH
	DNA_methylase		DNA_methylase		DNA_methylase
223320_PRF0	Pep_M12B_propep	**215690R.t2**		132640F.t1	Pep_M12B_propep
	Reprolysin		Reprolysin		Reprolysin
	TSP_1		TSP_1		TSP_1
	ADAM_spacer1		ADAM_spacer1		ADAM_spacer1
	TSP_1		TSP_1		TSP_1
	TSP_1		TSP_1		TSP_1
	TSP_1		TSP_1		TSP_1
	TSP_1		TSP_1		TSP_1
			TSP_1		TSP_1
			TSP_1		TSP_1
			TSP_1		TSP_1

**227800_PRF0**	SRCR	**010500R.t1**	SRCR	**283050F.t3**	SRCR
	SRCR		SRCR		SRCR
	SRCR		SRCR		SRCR
	Lysyl_oxidase		Lysyl_oxidase		Lysyl_oxidase
**237250_PRF0**		111050F.t1*	A2M_N	171620R.t1*	A2M_N
			A2M_N_2		A2M_N_2
			ANATO		ANATO
			A2M		A2M
			Thiol-ester_cl		Thiol-ester_cl
			A2M_comp		A2M_comp
	A2M_recep		A2M_recep		A2M_recep
	NTR		NTR		NTR
**237260_PRF0**	A2M_N	111050F.t1*	A2M_N	171620R.t1*	A2M_N
	A2M_N_2		A2M_N_2		A2M_N_2
	ANATO		ANATO		ANATO
	A2M		A2M		A2M
	Thiol-ester_cl		Thiol-ester_cl		Thiol-ester_cl
	A2M_comp		A2M_comp		A2M_comp
			A2M_recep		A2M_recep
			NTR		NTR
246010_PRF0*	PG_binding_1	098400F.t1*	PG_binding_1	**217220R.t1**	PG_binding_1
	Peptidase_M10		Peptidase_M10		Peptidase_M10
	Hemopexin		Hemopexin		Hemopexin
	Hemopexin		Hemopexin		Hemopexin
	Hemopexin		Hemopexin		Hemopexin
	Hemopexin		Hemopexin		Hemopexin
					MFS_1
247370_PRF0*	WIF	113360F.t1*	WIF	045780R.t1*	WIF
	Pkinase_Tyr		Pkinase_Tyr		Pkinase_Tyr
248080_PRF0	EGF_3	073650R.t1	EGF_3	246540R.t1	EGF_3
	Sushi		Sushi		Sushi
	Sushi		Sushi		Sushi
	VWA		VWA		VWA
	Trypsin		Trypsin		Trypsin
253950_PRF0*		**290630R.t1**	Histone	135030R.t1*	
			RhoGEF		
	Histone		Histone		Histone
	RhoGEF		RhoGEF		RhoGEF
	PH		PH		PH
	RasGEF_N		RasGEF_N		RasGEF_N
	RasGEF		RasGEF		RasGEF
253960_PRF0*	zf-Sec23_Sec24	290640R.t1*	zf-Sec23_Sec24	135040R.t1*	zf-Sec23_Sec24
	Sec23_trunk		Sec23_trunk		Sec23_trunk
	Sec23_BS		Sec23_BS		Sec23_BS
	Sec23_helical		Sec23_helical		Sec23_helical
	Gelsolin		Gelsolin		Gelsolin
254170_PRF0*	ABC_membrane	154120R.t1*	ABC_membrane	026920R.t1*	ABC_membrane
	ABC_tran		ABC_tran		ABC_tran
	ABC_membrane		ABC_membrane		ABC_membrane
	ABC_tran		ABC_tran		ABC_tran
**256520_PRF0**		044700R.t1*	Myb_DNA-binding	105830R.t1*	Myb_DNA-binding
			SWIRM		SWIRM

	JAB		JAB		JAB
**256530_PRF0**	Myb_DNA-binding	044700R.t1*	Myb_DNA-binding	105830R.t1*	Myb_DNA-binding
	SWIRM		SWIRM		SWIRM
			JAB		JAB
257610_PRF0*	EST1	174840F.t1*	EST1	039650R.t1*	EST1
	EST1_DNA_bind		EST1_DNA_bind		EST1_DNA_bind
	EST1_DNA_bind		EST1_DNA_bind		EST1_DNA_bind
	PIN_4		PIN_4		PIN_4
**258810_PRF0**	Glyco_transf_29	**000220F.t1**	Glyco_transf_29	299600F.t1*	Glyco_transf_29
	Glyco_transf_29		Glyco_transf_29		
258870_PRF0*	STAT_int	049290R.t1*	STAT_int	154380F.t1*	STAT_int
	STAT_alpha		STAT_alpha		STAT_alpha
	STAT_bind		STAT_bind		STAT_bind
	SH2		SH2		SH2
259020_PRF0*	GRAM	011870R.t1*	GRAM	075250R.t1*	GRAM
	GRAM		GRAM		GRAM
	RabGAP-TBC		RabGAP-TBC		RabGAP-TBC
272770_PRF0*	AAA_11	091600R.t1*	AAA_11	182450R.t1*	AAA_11
	AAA_11		AAA_11		AAA_11
	AAA_12		AAA_12		AAA_12
283240_PRF0	Reeler	241240R.t1	Reeler	179850R.t1	Reeler
	Spond_N		Spond_N		Spond_N
	fn3		fn3		fn3
	TSP_1		TSP_1		TSP_1
	fn3		fn3		fn3
	TSP_1		TSP_1		TSP_1
	TSP_1		TSP_1		TSP_1
293630_PRF0*		**312700R.t1**	TPR_12	110520R.t2*	
			TPR_12		
			TPR_7		
			TPR_12		
			TPR_7		
			TPR_12		
	Chorein_N		Chorein_N		Chorein_N
	ATG_C		ATG_C		ATG_C
**293740_PRF0**		**151150R.t1**	SHR-BD	**257130F.t1**	
			VPS13_C		
	SHR-BD		SHR-BD		SHR-BD
	VPS13_C		VPS13_C		VPS13_C
**209920_PRM0**	UBA	**151150R.t1**		**257120F.t1**	UBA
					UBA
			SHR-BD		
			VPS13_C		
			SHR-BD		
			VPS13_C		
**209940_PRF0**	Chorein_N	**151170R.t**1		**257100F.t1**	
	VPS13		VPS13		VPS13
	VPS13_mid_rpt		VPS13_mid_rpt		VPS13_mid_rpt
321850_PRF0*	ANF_receptor	**030780R.t1**		275750F.t1*	ANF_receptor
	Lig_chan-Glu_bd		Lig_chan-Glu_bd		Lig_chan-Glu_bd
	Lig_chan		Lig_chan		Lig_chan

319230_PRF0*		**102020R.t1**	cNMP_binding	158680R.t1*	
	cNMP_binding		cNMP_binding		cNMP_binding
	DEP		DEP		DEP
	cNMP_binding		cNMP_binding		cNMP_binding
	RasGEF_N				RasGEF_N
	RasGEF				RasGEF
			SEA		
			SEA		
325460_PRF0	FG-GAP	100710F.t1	FG-GAP	196740F.t1	FG-GAP
	FG-GAP		FG-GAP		FG-GAP
	Integrin_alpha2		Integrin_alpha2		Integrin_alpha2
**320370_PRF0**	zf-RING_5	**145570R.t1**		**031850F.t1**	zf-RING_5
			zf-C3HC4_3		
	zf-B_box		zf-B_box		zf-B_box
	fn3		fn3		fn3
	SPRY				
325780_PRF0*		**100260F.t1**	Glycos_transf_2	**196320F.t1**	
			BACK		
			Glycos_transf_2		Glycos_transf_2
			Ricin_B_lectin		Ricin_B_lectin
	Glycos_transf_2		Glycos_transf_2		Glycos_transf_2
	Ricin_B_lectin		Ricin_B_lectin		Ricin_B_lectin
**328450_PRF0**		212930R.t1*	WAP	040720F.t1*	WAP
			Kazal_2		Kazal_2
			Kunitz_BPTI		Kunitz_BPTI
	Kunitz_BPTI		Kunitz_BPTI		Kunitz_BPTI
	NTR		NTR		NTR
**328460_PRF0**	WAP	212930R.t1*	WAP	040720F.t1*	WAP
	Kazal_2		Kazal_2		Kazal_2
	Kunitz_BPTI		Kunitz_BPTI		Kunitz_BPTI
	Kunitz_BPTI		Kunitz_BPTI		Kunitz_BPTI
			NTR		NTR
**328470_PRF0**		**212960R.t3**	ABC_membrane	**040670F.t2**	
	ABC_membrane		ABC_membrane		ABC_membrane
	ABC_tran		ABC_tran		ABC_tran
	ABC_membrane		ABC_membrane		ABC_membrane
	ABC_tran				
			ABC_membrane		ABC_membrane
	ABC_membrane		ABC_membrane		
	ABC_tran				
			ABC_membrane		
	ABC_membrane		ABC_membrane		
	ABC_tran		ABC_tran		
**328810_PRF0**		**279190F.t1**	zf-HC5HC2H	**172210F.t4**	
			zf-HC5HC2H		zf-HC5HC2H
			PHD		PHD
			PHD		PHD
			PHD		PHD
	zf-HC5HC2H		zf-HC5HC2H		zf-HC5HC2H
	FYRN		FYRN		FYRN
	FYRC		FYRC		FYRC
	FYRC				
	SET		SET		SET
**328940_PRF0**	dsrm	**278970F.t1**	dsrm	**172050F.t2**	

	dsrm<br>A_deamin<br>A_deamin		dsrm<br>A_deamin<br><br>adh_short		dsrm<br>A_deamin
329040_PRF0*	Flavodoxin_1<br>FAD_binding_1<br>NAD_binding_1	278780F.t1*	Flavodoxin_1<br>FAD_binding_1<br>NAD_binding_1	171870F.t1*	Flavodoxin_1<br>FAD_binding_1<br>NAD_binding_1
329370_PRF0*	PLAT<br>Lipoxygenase	**246260F.t1**	PLAT<br>Lipoxygenase<br>Lipoxygenase	286940R.t1*	PLAT<br>Lipoxygenase
329970_PRF0	DAG_kinase_N<br>C1_1<br>C1_1<br>DAGK_cat<br>DAGK_acc	242250R.t1	DAG_kinase_N<br>C1_1<br>C1_1<br>DAGK_cat<br>DAGK_acc	057640F.t1	DAG_kinase_N<br>C1_1<br>C1_1<br>DAGK_cat<br>DAGK_acc
330040_PRF0*	Activin_recp<br>Pkinase	242390R.t1*	Activin_recp<br>Pkinase	**057490F.t2**	Activin_recp<br>Pkinase<br>Activin_recp<br>Pkinase
330210_PRF0*	EF-hand_1<br>Mito_carr<br>Mito_carr<br>Mito_carr	242710R.t1*	EF-hand_1<br>Mito_carr<br>Mito_carr<br>Mito_carr	057220F.t1*	EF-hand_1<br>Mito_carr<br>Mito_carr<br>Mito_carr

Orthologs are absent in the Swiss-Prot database

000200_PFF0	V-set	094410F.t1	V-set	144580F.t1	V-set
	CBM_14		CBM_14		CBM_14
001280_PFF0	CBM_14	259500R.t1	CBM_14	169170F.t1	CBM_14
	ILEI		ILEI		ILEI
017700_PFF0		002350R.t1	zf-B_box	204650R.t1	zf-B_box
	Gal_Lectin		Gal_Lectin		Gal_Lectin
	Gal_Lectin		Gal_Lectin		Gal_Lectin
	GPS		GPS		GPS
	7tm_2		7tm_2		7tm_2
018300_PFF0	Lectin_C	261420F.t1		222240F.t1	Lectin_C
	fn2				fn2
	fn2				fn2
	fn2		fn2		fn2
	fn2		fn2		fn2
	fn2		fn2		fn2
040970_PFF0	kringle	236680F.t1	kringle	076590R.t1	kringle
	kringle		kringle		kringle
	trypsin		trypsin		trypsin
068220_PFF0	fn2	288190R.t1	fn2	100050R.t1	fn2
	fn2		fn2		fn2
	fn2		fn2		fn2
	fn2		fn2		fn2
	CUB		CUB		CUB
	CUB		CUB		CUB
080050_PFF0	N-glycanase_N	072890R.t1	N-glycanase_N	251010F.t1	N-glycanase_N
	N-glycanase_C		N-glycanase_C		N-glycanase_C
124390_PFF0	cEGF	140980F.t1	cEGF	141260F.t1	cEGF
	FXa_inhibition		FXa_inhibition		FXa_inhibition
	EGF_CA		EGF_CA		EGF_CA
	EGF_CA		EGF_CA		EGF_CA
	NIDO		NIDO		NIDO
			VWD		VWD
	EGF_CA		EGF_CA		EGF_CA
	EGF_CA		EGF_CA		EGF_CA
			cEGF		cEGF
	SEA		SEA		SEA
	EGF_CA		EGF_CA		EGF_CA
159900_PFF0	AAA_8	101480R.t1	AAA_8	158030R.t1	AAA_8
	MT		MT		MT
	AAA_9		AAA_9		AAA_9
			AAA_9		AAA_9
	Dynein_heavy		Dynein_heavy		Dynein_heavy
167280_PRF0	Kringle	007220R.t1	Kringle	118250R.t1	Kringle
	Somatomedin_B		Somatomedin_B		Somatomedin_B
	7tm_2		7tm_2		7tm_2
173360_PRF0	Astacin	299040F.t1	Astacin	053640F.t1	Astacin
	kringle		kringle		kringle

180420_PRF0	Somatomedin_B	050300R.t1	Somatomedin_B	069800F.t1	Somatomedin_B
	7tm_2		7tm_2		7tm_2
184730_PRF0		002500F.t1		176740F.t1	Ldl_recept_a
	Ldl_recept_a		Ldl_recept_a		Ldl_recept_a
	Kringle		Kringle		Kringle
	Kringle		Kringle		Kringle
					Somatomedin_B
	7tm_2		7tm_2		7tm_2
188610_PRF0	ASC	021920R.t1	ASC	155310R.t1	ASC
	F5_F8_type_C		F5_F8_type_C		
	F5_F8_type_C		F5_F8_type_C		F5_F8_type_C
	Kringle		Kringle		Kringle
	ASC		ASC		ASC
196740_PRF0	Fibrinogen_C	024030F.t2	Fibrinogen_C	086240F.t1	Fibrinogen_C
	Fibrinogen_C		Fibrinogen_C		Fibrinogen_C
	I-set		I-set		I-set
	fn3		fn3		fn3
	fn3		fn3		fn3
	fn3		fn3		fn3
	fn3		fn3		fn3
	Y_phosphatase		Y_phosphatase		Y_phosphatase
	Y_phosphatase		Y_phosphatase		Y_phosphatase
200810_PRF0	Astacin	016310R.t1	Astacin	104370F.t1	Astacin
	CUB		CUB		CUB
	MAM		MAM		MAM
258300_PRF0		060660R.t1	SRCR	006090R.t1	SRCR
			Kringle		Kringle
			Kringle		Kringle
			Ldl_recept_a		Ldl_recept_a
			PAN_1		PAN_1
	Ldl_recept_a		Ldl_recept_a		Ldl_recept_a
	SRCR		SRCR		SRCR
	Ldl_recept_a		Ldl_recept_a		Ldl_recept_a
	Trypsin		Trypsin		Trypsin
260260_PRF0	BACK	162090R.t1	BACK	152800F.t1	BACK
	Kelch_1		Kelch_1		Kelch_1
269420_PRF0		303590R.t1	Death	009080F.t1	Death
			CUB		CUB
	CUB		CUB		CUB
	Ldl_recept_a		Ldl_recept_a		Ldl_recept_a
	Fz		Fz		Fz
	Kringle		Kringle		Kringle
	Neur_chan_memb		Neur_chan_memb		Neur_chan_memb
304470_PRF0	Ldl_recept_a	092860F.t1	Ldl_recept_a	002980R.t1	Ldl_recept_a
	VWA		VWA		VWA
	Zona_pellucida		Zona_pellucida		Zona_pellucida
320550_PRF0	Kringle	145290F.t1		009580R.t1	Kringle
	Kringle				Kringle
	Lectin_C				Lectin_C
	CUB				CUB
	Lectin_C		Lectin_C		Lectin_C
	MAM		MAM		MAM

	Lectin_C<br>Kringle<br>SRCR		Lectin_C<br>Kringle<br>SRCR		Lectin_C<br>Kringle<br>SRCR
329690_PRF0	BTB<br>BTB<br>BACK<br>Kelch_1<br>Kelch_1<br>Kelch_1<br>Kelch_1	245850F.t1	BTB<br>BTB<br>BACK<br>Kelch_1<br>Kelch_1<br>Kelch_1<br>Kelch_1	287390R.t1	BTB<br>BTB<br>BACK<br>Kelch_1<br>Kelch_1<br>Kelch_1<br>Kelch_1
329890_PRF0	COR<br>Death	047410F.t1	COR<br>Death	173700F.t1	 Death
